# Self-Reported Exposure to Secondhand Smoke and Support for Complete Smoking Bans in Multiunit Housing Among Smokers in the United States, Canada, and the United Kingdom

**DOI:** 10.5888/pcd17.200201

**Published:** 2020-11-25

**Authors:** Pete Driezen, Geoffrey T. Fong, Andrew Hyland, Lorraine V. Craig, Genevieve Sansone, Sara C. Hitchman, K. Michael Cummings

**Affiliations:** 1 University of Waterloo, Waterloo, Ontario, Canada; 2 Ontario Institute for Cancer Research, Toronto, Ontario, Canada; 3 Roswell Park Comprehensive Cancer Center, Buffalo, New York; 4 King’s College London, London, United Kingdom; 5 Medical University of South Carolina, Charleston, South Carolina

## Abstract

**Introduction:**

Involuntary exposure to secondhand smoke most frequently occurs at home, which is problematic for residents of multiunit housing (MUH). The primary objective of this study was to estimate the extent of secondhand smoke incursions into the homes of MUH smokers who banned smoking in their homes but lived in buildings where smoking is allowed.

**Methods:**

We used data from Wave 9 of the International Tobacco Control Four Country Survey. We estimated 1) the prevalence of complete smoking bans among smokers living in single-family homes vs MUH in the United States (n = 3,208), Canada (n = 1,592), and the United Kingdom (n = 1,403) from 2013 to 2015; 2) the extent of secondhand smoke incursions into the homes of MUH smokers who banned smoking in their units but lived in buildings that allow smoking; and 3) MUH smokers’ preferences for complete smoking bans in MUH. Weighted multivariable logistic regression estimated the country-specific adjusted prevalence of all outcomes.

**Results:**

Overall, 53.0% of smokers living in single-family homes completely banned smoking in their homes, compared with 44.8% of smokers in MUH. Across all 3 countries, only 27.8% of MUH smokers reported that smoking was completely prohibited in their building. A similar percentage of MUH smokers who banned smoking in their home but lived in buildings allowing smoking reported a secondhand smoke incursion into their home in the United States (29.9%; 95% CI, 20.4%–41.5%), Canada (38.4%; 95% CI, 26.7%–51.6%), and the United Kingdom (24.7%; 95% CI, 15.7%–36.7%). Across all 3 countries, 36.1% (95% CI, 33.4%–38.9%) of smokers in MUH reported they preferred a complete smoking ban in all building areas.

**Conclusion:**

A need remains to educate MUH operators and residents about the benefits of comprehensive smoke-free policies.

**Summary**

**What is already known about this topic?**

Smoking in multiunit housing involuntarily exposes residents to secondhand smoke via transfer from other units.

**What is added by this report?**

In this first international comparative study of smoking in multiunit housing, 30%, 38%, and 25% of smokers in the United States, Canada, and the United Kingdom, respectively, who prohibited smoking in their homes but lived in multiunit buildings that allowed smoking, reported secondhand smoke incursions into their homes in 2013–2015.

**What are the implications for public health practice?**

Protecting multiunit housing residents from secondhand smoke remains a challenge in the United States, Canada, and the United Kingdom. Policies and practices must be developed and implemented to reduce this hazard.

## Introduction

As smoke-free policies become more common in bars, restaurants, and workplaces, most involuntary exposure to secondhand smoke (SHS) occurs in the home (1,2). Exposure to SHS is causally linked to lung cancer, stroke, and cardiovascular disease in adults and low birth weight, sudden infant death syndrome, ear infections, and asthma in children (3,4). In the United States, SHS exposure at home accounted for more than 358,000 excess emergency department visits among adult nonsmokers and 102,000 excess emergency department visits among children in 2010, resulting in more than $462 million (in 2014 dollars) in additional health care costs from avoidable emergency department visits alone (5,6). Despite the declining prevalence of involuntary exposure to SHS and the increasing prevalence of complete smoking bans in smokers’ homes (5,7,8), involuntary exposure to SHS at home remains an important public health problem (9).

In 2018, 80.5 million people in the United States lived in multiunit housing (MUH), 9% of whom lived in government-subsidized housing (10,11). Involuntary exposure is problematic for residents of MUH even if they prohibit smoking in their own homes because SHS readily infiltrates other units through walls, doors, windows, ductwork, and ventilation systems (12). As many as 29%, in 2011 (13), to 44%, in 2010 (14), of MUH residents in the United States reported SHS infiltrating their homes, although local estimates vary across US communities (15,16). Involuntary exposure also varies by housing type: a greater percentage of public and subsidized MUH residents report SHS incursions than do residents in privately owned MUH (17).

Most evidence on SHS incursions into MUH comes from the United States. Limited international data are available. In a 2010 study of 1 Canadian community in southern Ontario, 52% of MUH residents living in subsidized housing reported SHS infiltrating their homes (18). Also in 2010, 22% of MUH residents in Denmark reported an SHS incursion (19). From 2009 to 2011, 16% to 21% of nonsmoking MUH residents in Korea who prohibited smoking in their homes reported weekly SHS incursions (20). Although these findings are broadly consistent with US estimates, internationally comparable estimates are lacking.

Involuntary exposure to SHS is problematic for nonsmokers and smokers alike because smokers may ban smoking in their own homes to protect children and family members. The objectives of this study were to 1) compare the prevalence of home smoking bans among smokers living in single-family dwellings versus MUH in the United States, Canada, and the United Kingdom; 2) estimate the extent of SHS incursions among MUH smokers who banned smoking in their homes but lived in buildings allowing smoking; and 3) estimate support for complete smoking bans in MUH among current and former smokers living in MUH.

## Methods

Data for this study came from Wave 9 of the International Tobacco Control (ITC) Four Country Survey conducted from February 2013 to March 2015. The ITC Survey was a prospective cohort survey of nationally representative samples of smokers in the United States, Canada, the United Kingdom, and Australia. Beginning in 2002, the ITC Survey used a stratified sampling design to randomly select smokers in geographic strata in each country. Smokers lost to attrition were replaced in subsequent waves by newly recruited smokers using the same sampling design. Smokers who quit smoking were retained and followed over time. Sampling weights were computed for all respondents to ensure that estimates represented the population of smokers in each country at the time of the survey. Initial cooperation rates ranged from 79% in the United Kingdom to 83% in the United States. Details of the methods of the ITC Four Country Survey are available elsewhere (21–23).

In Wave 9, data were collected using both telephone and web-based interviewing methods from 3,208 current and former smokers in the United States, 1,592 in Canada, and 1,403 in the United Kingdom. We excluded respondents in Australia because questions on smoking in MUH were not asked in the Wave 9 Australia Survey. All survey protocols and materials, including the survey questionnaires, were cleared for ethics by the Office of Research Ethics, University of Waterloo, in Canada, and the Office of Research Subject Protection, Roswell Park Comprehensive Cancer Center, in the United States.

### Measures

#### Multiunit housing

In the Wave 9 ITC Survey, current and former smokers were asked to indicate whether they currently lived in single-family homes or multiunit dwellings (in Canada and the United States, defined as a semidetached house, a townhouse, or an apartment building; in the United Kingdom, a semidetached house, a terraced house, a conversion, a flat, or a quarter villa). Respondents who reported living in a single-family home were further asked whether that dwelling included more than 1 household or residence, such as an apartment in the basement or attic, or where someone is renting a room. Respondents who reported they lived in a multiunit building or a single-family home with multiple households or residences were classified as living in MUH. Respondents were not asked to specify whether they lived in market-rate or subsidized housing.

#### Outcome measures

Both residents of single-family homes and MUH were asked to describe personal rules about smoking in their homes or units. MUH residents were also asked about official building policies on smoking in indoor areas and whether SHS entered their units from somewhere inside or outside their building. First, all respondents were asked to describe smoking in their homes. Responses were “smoking is allowed anywhere,” “smoking is *never* allowed *anywhere*,” and “something in between.” Responses were dichotomized into “smoking is never allowed” (complete home ban) and “smoking is allowed” (“smoking is allowed anywhere” and “something in between”). Second, MUH respondents were asked about their building’s official smoking policy for indoor areas; responses were categorized as complete bans (“smoking is prohibited in all indoor areas of the building, including individual residences”), partial/no bans (“smoking is prohibited in shared indoor areas, but allowed inside individual residences,” and “smoking is allowed anywhere”), and “don’t know.”

Third, MUH residents were asked about SHS incursions into their units using the question, “How often, if at all, does tobacco smoke enter your own residential unit from somewhere else inside or outside your building?” Respondents who reported noticing tobacco smoke “less than once a week,” “1 to 3 days a week,” “4 to 6 days a week,” or “every day” were classified as reporting “any incursion” into their units, and those who reported “never” were classified as “no incursions reported.” Responses were further dichotomized into 1) at least weekly incursions versus otherwise and 2) daily incursions versus otherwise. Finally, preferences for smoke-free building policies were assessed by asking respondents whether they preferred a policy in their building prohibiting smoking in “*all* areas, including individual residences, common areas, *and* exterior grounds.” Responses were “strongly prefer,” “somewhat prefer,” “slightly prefer,” and “would *not* prefer”. Responses were dichotomized as any preference (strongly + somewhat + slightly) versus no preference.

#### Sociodemographic measures

Several sociodemographic measures assessed the characteristics of respondents participating in the ITC Wave 9 Survey. They were sex (male and female), age (18–24, 25–39, 40–54, and ≥55), race (white and nonwhite, which included Black/African American/Black British, Native American/First Nation, Hispanic/Latino/Latin American, Asian [Chinese, Japanese, Korean, Southeast Asian, West Asian]/Asian British, Pacific Islander/Filipino, and other, including mixed race), marital status (single, married/common-law/de facto, and divorced/widowed/separated) and whether children were living in the home (no children/no children in home and ≥1 child). Both education and income were classified into low, moderate, and high, with an additional “not stated” category for income. In the United States and Canada, low level of education was defined as having a high school education or less, moderate level of education was defined as having completed technical/trade school/community college or having some university education, and high level of education was defined as having completed a university degree or a postgraduate degree. In the United Kingdom, low level of education was defined as having secondary or vocational training or lower, moderate level of education was defined as having some college or university but no degree, and high level of education was defined as having completed a university degree or postgraduate degree.

Income classification also differed by country. In Canada and the United States, respondents having annual household incomes below $30,000 were classified as low income and respondents having annual household incomes from $30,000 to $59,999 were classified as moderate income. All respondents having annual household incomes of $60,000 or higher were classified as high income. In the United Kingdom, respondents having annual household incomes of £15,000 or less were classified as low income and respondents having annual household incomes from £15,001 to £30,000 were classified as moderate income. All respondents having annual household incomes greater than £30,000 were classified as high income.

Two final measures were used to account for differences in survey sampling and survey administration across countries: 1) respondents recruited before Wave 9 versus respondents recruited in Wave 9 and 2) respondents surveyed by telephone versus respondents surveyed online.

#### Smoking behaviors

Respondents were classified into 3 groups based on their smoking behaviors: daily smokers, nondaily smokers (smoke on at least a monthly basis), and former smokers. Former smokers reported having quit either within the previous 12 months or more than 12 months ago. In regression models, daily and nondaily smokers were combined into a single group, and hereinafter, the term “smokers” refers to both current and former smokers.

### Statistical analysis

We used SAS-callable SUDAAN (SAS version 9.4, SAS Institute, Inc; SUDAAN version 11.0.3, RTI International) to account for the stratified sampling design and sampling weights. We estimated descriptive statistics to describe the characteristics of smokers in each country. We then used binary logistic regression to estimate the adjusted percentage of smokers in each country who completely banned smoking in their homes by housing type (single-family home vs MUH). These adjusted percentages, or average marginal effects, represent the weighted average of the predicted probabilities for each country across all levels of all covariates included in the model. Thus, these adjusted percentages account for differences in covariate distributions across countries and are the regression-based equivalent of epidemiologic standardization methods (24,25). All adjusted percentages controlled for sex, age group, smoking status, income, education, whether children lived in the home, wave of recruitment, and survey mode.

Additional binary or multinomial logistic models were fit to estimate 1) the country-specific percentage of smokers who reported that smoking was completely banned (complete ban, no ban/partial ban vs don’t know) in their building among the subset of smokers living in MUH, 2) the country-specific percentage of smokers who prohibited smoking in their own units among the subset of smokers living in MUH where smoking was allowed, and 3) the country-specific percentage of smokers reporting SHS incursions into their units among the subset of smokers having personal smoking bans but living in MUH where smoking was allowed. Three types of SHS incursions were estimated: 1) any incursion (less than once per week or more frequently vs never), 2) weekly incursions (at least once per week vs otherwise), and 3) daily incursions (daily vs otherwise). A final multinomial model was fit to estimate the country-specific percentage of MUH smokers who would slightly prefer, somewhat prefer, strongly prefer, or would not prefer living in a smoke-free building. For all estimated regression models, we used a Wald χ^2^ test to test the overall effect of country and *P* < .05 was used to denote significant differences. We also tested differences in predicted marginal estimates between countries, and we used a Bonferroni correction to account for multiple testing. Regression models satisfied appropriate diagnostic checks (no evidence of multicollinearity for all models, Hosmer–Lemeshow goodness-of-fit tests for binary logistic models).

## Results

Key differences between the countries included the racial composition and income distribution of smokers. A greater percentage of smokers in the United States than in Canada and the United Kingdom were nonwhite (21.3%, 9.4%, 8.4%, respectively) and in the low-income group (38.2%, 19.8%, 28.0%, respectively) (Table 1). Although a minority of smokers in the United States (32.0%) and Canada (43.2%) lived in MUH, a majority (72%) of smokers in the United Kingdom lived in MUH.

**Table 1:** Characteristics of Current and Former Smokers (N = 6,203) in the United States, Canada, and the United Kingdom, by Country, 2013–2015^a^

Characteristic	United States (n = 3,208)		Canada (n = 1,592)		United Kingdom (n = 1,403)	
	n	% (95% CI)	n	% (95% CI)	n	% (95% CI)
**Sex**						
Male	1,550	54.1 (51.8–56.5)	749	56.6 (53.6–59.6)	678	51.9 (48.5–55.3)
Female	1,658	45.9 (43.5–48.2)	843	43.4 (40.4–46.4)	725	48.1 (44.7–51.5)
**Age group**						
18–24	185	11.6 (9.7–13.8)	60	8.1 (6.1–10.7)	71	13.3 (10.0–17.3)
25–39	700	31.8 (29.5–34.3)	337	31.6 (28.6–34.8)	340	29.6 (26.7–32.8)
40–54	1,015	30.4 (28.3–32.5)	688	36.0 (33.3–38.8)	562	31.2 (28.5–34.0)
≥55	1,308	26.2 (24.5–28.0)	507	24.3 (22.0–26.7)	430	25.9 (23.4–28.6)
**Race^b^**						
White	2,486	78.7 (76.6–80.5)	1,472	90.6 (88.3–92.5)	1287	91.6 (89.3–93.4)
Nonwhite	716	21.3 (19.5–23.4)	119	9.4 (7.5–11.7)	107	8.4 (6.6–10.7)
**Marital status^b^**						
Single	650	25.9 (23.7–28.2)	321	22.1 (19.5–24.9)	320	26.2 (23.1–29.5)
Married/common-law/de facto	1,724	51.8 (49.4–54.2)	921	61.4 (58.4–64.4)	736	53.6 (50.2–57.0)
Divorced/widowed/separated	827	22.4 (20.5–24.3)	344	16.5 (14.5–18.6)	338	20.2 (18.0–22.7)
**Any children living in home^b^**						
No children or no children in home	2,332	67.3 (65.0–69.6)	1,207	70.8 (67.7–73.7)	1,052	70.9 (67.5–74.0)
At least 1 child	869	32.7 (30.4–35.0)	381	29.2 (26.3–32.3)	345	29.1 (26.0–32.5)
**Education^b^**						
Low	1,277	41.4 (39.1–43.8)	611	36.9 (34.0–39.9)	667	46.6 (43.2–50.0)
Moderate	1,258	37.3 (35.1–39.6)	629	41.1 (38.0–44.2)	388	28.0 (25.1–31.2)
High	673	21.3 (19.3–23.4)	344	22.0 (19.6–24.7)	334	25.4 (22.5–28.5)
**Income**						
Low	1,196	38.2 (35.9–40.6)	356	19.8 (17.5–22.3)	429	28.0 (25.3–30.8)
Moderate	938	28.0 (25.9–30.1)	544	34.7 (31.8–37.7)	422	29.4 (26.4–32.6)
High	1,005	31.6 (29.4–33.9)	545	36.9 (34.0–39.9)	434	34.2 (30.9–37.6)
Not reported	69	2.3 (1.6–3.1)	147	8.5 (7.0–10.4)	118	8.4 (6.7–10.4)
**Housing type^b^**						
Single-family home	2,176	68.0 (65.6–70.2)	962	56.8 (53.7–59.7)	393	28.0 (25.0–31.2)
Multiunit housing	904	32.0 (29.8–34.4)	611	43.2 (40.3–46.3)	989	72.0 (68.8–75.0)
**Smoking status**						
Daily smoker	2,199	67.0 (64.6–69.3)	1,128	69.1 (66.1–72.0)	1,018	72.8 (69.6–75.8)
Non-daily smoker	412	13.0 (11.5–14.6)	81	5.2 (3.9–6.9)	85	5.4 (4.3–6.8)
Former smoker	597	20.1 (18.0–22.3)	383	25.7 (23.0–28.6)	300	21.8 (18.9–24.9)
**Cigarettes smoked per day^c^**						
Sample, n	2,140	—	1,121	—	1,010	—
Mean no. of cigarettes	—	15.2 (14.7–15.7)	—	18.1 (17.4–18.7)	—	16.3 (15.7–16.9)
**Duration since quitting^d^**						
Within last 12 months	392	63.8 (57.2–69.9)	84	20.4 (15.9–25.8)	77	29.0 (21.9–37.3)
More than 12 months ago	205	36.2 (30.1–42.8)	299	79.6 (74.2–84.1)	223	71.0 (62.7–78.1)

a Data source: Wave 9 of the International Tobacco Control Four Country Survey conducted from February 2013 to March 2015. All values are n (% [95% CI]) except for category “cigarettes smoked per day.”

b Sample sizes in these categories do not sum to the country total because respondents could choose not to answer these questions.

c Among daily smokers only.

d Among former smokers only.

### Complete home smoking bans

On average, from 2013 to 2015, 53.0% of smokers living in single-family homes reported they completely banned smoking in their home, compared with only 44.8% of smokers living in MUH (Table 2). We observed similar differences in all 3 countries. A significantly greater percentage of MUH smokers in the United States (48.8%) and Canada (44.7%) than in the United Kingdom (35.8%) reported completely banning smoking in their homes (United States vs United Kingdom, Bonferroni *P* < .001; Canada vs United Kingdom, Bonferroni *P* = .02).

**Table 2:** Adjusted Percentage^a^ of Smokers in the United States, Canada, and the United Kingdom Who Completely Banned Smoking in Their Homes, by Type of Housing, 2013–2015^b^

Type of housing	United States (n = 3,032)		Canada (n = 1,553)		United Kingdom (n = 1,347)		Overall (n = 5,932)	
	n	% (95% CI)	n	% (95% CI)	n	% (95% CI)	n	% (95% CI)
Multiunit housing	872	48.8^c^ (44.6 to 53.1)	596	44.7^c^ (40.2 to 49.2)	963	35.8^d^ (32.1 to 39.6)	2,431	44.8 (42.1 to 47.4)
Single-family homes	2,160	55.8 (53.0 to 58.6)	957	51.7 (47.8 to 55.4)	384	48.1 (42.5 to 53.8)	3,501	53.0 (50.7 to 55.2)
Marginal difference	—	−7.0^e^ (−11.9 to −2.0)	—	−7.0 (−12.7 to −1.2)	—	−12.3^f^ (−19.1 to −5.6)	—	−8.2 (−11.6 to −4.8)

Abbreviation: —, does not apply.

a Adjusted percentages were estimated by using multivariable weighted logistic regression controlling for sex, age group, smoking status, income, education, children living in the home, wave of recruitment, and survey mode. The model included a country × housing type interaction effect (Wald χ_2_^2^ = 2.1; *P* = .35) to estimate the adjusted percentage of smokers who completely banned smoking in their homes in each type of housing across all countries.

b Data source: Wave 9 of the International Tobacco Control Four Country Survey conducted from February 2013 to March 2015.

c The percentage of smokers who completely banned smoking in their home was not significantly different between the United States and Canada.

d The percentage of smokers who completely banned smoking in their homes was significantly lower in the United Kingdom than in the United States (Bonferroni *P* < .001) and in Canada (Bonferroni *P* = .02).

e Difference between multiunit housing and single-family housing in the United States was significantly different (Bonferroni *P* = .04).

f Difference between multiunit housing and single-family housing in the United Kingdom was significantly different (Bonferroni *P* = .002).

### Rules on smoking in multiunit housing

**Figure 1:**
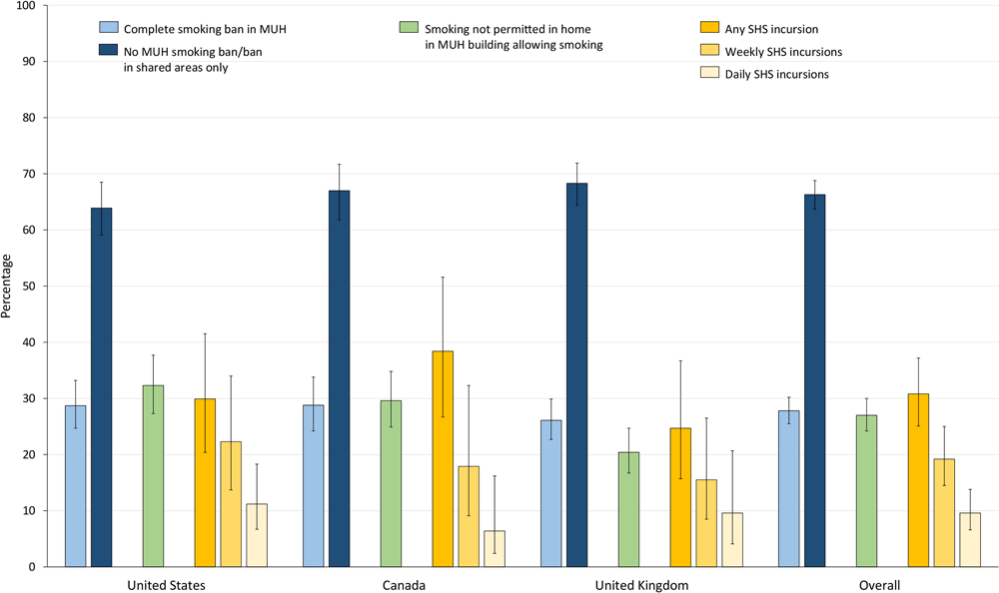
Percentage of smokers and former smokers living in multiunit housing (MUH) whose buildings have smoking bans and who are exposed to secondhand smoke (SHS) in their homes, 2013–2015, by country. Percentages for “complete smoking ban in MUH” and “no MUH smoking ban/ban in shared areas only” were based on a subset of current and former smokers who lived in MUH in each country in 2013–2015 (n = 2,446); percentages were estimated by using a multinomial logistic regression model. Percentages for “smoking not permitted in home in MUH building allowing smoking” were based on the subset of current and former smokers who were living in MUH but whose building did not ban smoking completely (n = 1,632). Percentages for “any SHS incursion,” “weekly SHS incursions,” and “daily SHS incursions” were based on a subset of current and former smokers living in MUH where smoking was not banned but who had complete smoking restrictions in their own homes (n = 393). All remaining percentages were estimated using logistic regression. All percentages were adjusted for sex, age group, smoking status, income, education, children living in the home, wave of recruitment, and survey mode. Error bars indicate 95% CIs.

Smokers living in MUH reported their building’s smoking policy (Figure 1). Across all countries, 27.8% (95% CI, 25.5%–30.2%) of MUH smokers reported that smoking was completely banned in their building, whereas 66.3% (95% CI, 63.7%–68.8%) reported their building had no ban or banned smoking only in shared areas. We found no significant differences by country (Wald χ_4_^2^ = 3.4; *P* = .49). Overall, 27.0% (95% CI, 24.2%–30.0%) of MUH smokers living in buildings where smoking was allowed reported they prohibited smoking in their own units. This percentage differed significantly across countries (Wald χ_2_^2^ = 15.4; *P* < .001). Of MUH smokers living in buildings allowing smoking, 32.3% (95% CI, 27.3%–37.7%) in the United States, 29.6% (95% CI, 24.9%–34.8%) in Canada, and 20.4% (95% CI, 16.7%–24.7%) in the United Kingdom reported complete smoking bans (United States vs United Kingdom, Bonferroni *P* < .001; Canada vs United Kingdom, Bonferroni *P* = .009).

**Table:** 

Country	Complete ban, % (95% CI)	No ban/ban in shared areas only, % (95% CI)	Smoking not permitted in home, % (95% CI)	Any SHS incursion, % (95% CI)	Weekly SHS incursions, % (95% CI)	Daily SHS incursions, % (95% CI)
United States	28.7 (24.7-33.2)	63.9 (59.1-68.5)	32.3 (27.3-37.7)	29.9 (20.4-41.5)	22.3 (13.7-34.0)	11.2 (6.7-18.3)
Canada	28.8 (24.2-33.8)	67.0 (61.8-71.7)	29.6 (24.9-34.8)	38.4 (26.7-51.6)	17.9 (9.1-32.3)	6.4 (2.4-16.2)
United Kingdom	26.1 (22.7-29.9)	68.3 (64.4-71.9)	20.4 (16.7-24.7)	24.7 (15.7-36.7)	15.5 (8.5-26.5)	9.6 (4.1-20.7)
Overall	27.8 (25.5-30.2)	66.3 (63.7-68.8)	27.0 (24.2-30.0)	30.8 (25.1-37.2)	19.2 (14.5-25.0)	9.6 (6.6-13.8)

### Reported SHS incursions and preferences for complete smoking bans

**Figure 2:**
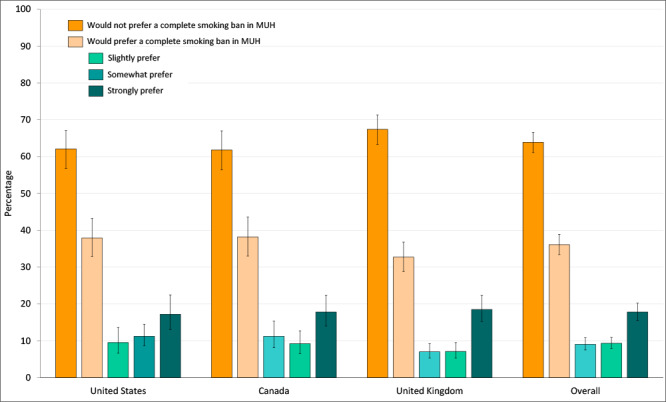
Preference for complete bans on smoking among smokers and former smokers living in multiunit housing (MUH) (n = 2,168) in the United States, Canada, and the United Kingdom, 2013–2015. “Slightly prefer,” “somewhat prefer,” and “strongly prefer” were combined to form the overall “would prefer a complete smoking ban in MUH” category. The overall percentage who “would prefer” complete bans was estimated by using logistic regression, whereas the percentage who would “slightly prefer,” “somewhat prefer,” and “strongly prefer” was estimated by using multinomial logistic regression. All percentages were adjusted for sex, age group, smoking status, income, education, children living in the home, wave of recruitment, and survey mode. Error bars indicate 95% CIs.

Among the subgroup of MUH smokers living in buildings where smoking was allowed but had banned smoking in their own units, 30.8% (95% CI, 25.1%–37.2%) reported any SHS incursion into their units, 19.2% (95% CI, 14.5%–25.0%) reported weekly incursions, and 9.6% (95% CI, 6.6%–13.8%) reported daily incursions (Figure 1). We found no significant differences across countries in the percentage of smokers reporting SHS incursions (Wald χ_2_^2^ = 2.7; *P* = .26 for any incursion; Wald χ_2_^2^ = 0.8, *P* = .66 for weekly incursions; and Wald χ_2_^2^ = 0.94; *P* = .62 for daily incursions). Among all MUH smokers, we also found no significant differences across countries in the percentage of smokers who reported preferring complete smoking bans in their building (Wald χ_2_^2^ = 3.9; *P* = .14). Overall, 36.1% (95% CI, 33.4%–38.9%) of smokers living in MUH reported they would slightly, somewhat, or strongly prefer a building policy prohibiting smoking in all areas, including individual areas, common areas, and exterior grounds (Figure 2).

**Table:** 

Country	Would not prefer	Would prefer	Slightly prefer	Somewhat prefer	Strongly prefer
United States	62.1 (56.8-67.1)	37.9 (32.9-43.2)	9.5 (6.6-13.6)	11.2 (8.6-14.5)	17.2 (13.0-22.4)
Canada	61.8 (56.4-67.0)	38.2 (33.0-43.6)	11.2 (8.1-15.3)	9.2 (6.5-12.7)	17.8 (14.0-22.3)
United Kingdom	67.4 (63.3-71.3)	32.7 (28.8-36.8)	7.0 (5.3-9.2)	7.1 (5.3-9.5)	18.5 (15.2-22.3)
Overall	63.9 (61.1-66.6)	36.1 (33.4-38.9)	9.0 (7.5-10.8)	9.3 (7.9-10.9)	17.8 (15.5-20.2)

## Discussion

Although most indoor public places in the United States, Canada, and the United Kingdom are now smoke-free, protecting MUH residents from SHS is a problem. From 2013 to 2015, 53.0% of smokers in the United States, Canada, and the United Kingdom living in single-family homes had completely banned smoking in their homes, compared with only 44.8% of smokers living in MUH. Almost two-thirds of MUH smokers reported that smoking was allowed either in some or all areas of their building. Because SHS transfers throughout MUH, a key challenge lies in preventing smokers from smoking inside MUH. This is essential because almost one-third of smokers living in MUH who prohibited smoking in their own homes still reported SHS incursions in their units in the 3 countries we studied. Moreover, only 36.1% of smokers living in MUH preferred that smoking be prohibited in all areas of their building. Comprehensive smoke-free MUH policies, either legislated by government or implemented voluntarily by MUH operators, can eliminate involuntary SHS exposure in MUH, producing health gains for all MUH residents.

Eliminating SHS from MUH requires support from private MUH operators and residents (14). Surveys of MUH operators indicate that owners and managers of MUH buildings would be motivated to implement smoke-free policies if evidence of demand existed for smoke-free units and if tenants requested these policies (26). Given the similarity of involuntary SHS incursions into MUH smokers’ homes in the United States, Canada, and the United Kingdom, an international effort in developing comprehensive smoke-free policies for MUH is crucial. The development of comprehensive smoke-free policies is especially important in countries where a large percentage of the population resides in MUH, as in the United Kingdom, where 60.8% of the population lived in semidetached houses in 2018 and another 14.8% lived in apartment buildings (27,28). In addition, it is necessary to ensure that no tenants be exempted from smoke-free policies. Kaufman et al demonstrated that exempting existing tenants from new smoke-free policies contributes to noncompliance with those policies (29). Finally, the increasing popularity of e-cigarettes poses new challenges to the implementation of smoke-free policies in MUH, because some e-cigarettes look like cigarettes, which may make policy compliance difficult to enforce (30).

Our study has several limitations. First, it relied on data from current smokers and former smokers only. Therefore, we could not estimate the extent of SHS exposure or preferences for complete bans among nonsmokers living in MUH. Second, estimates of SHS incursions are based on self-reported incursions and do not reflect objective atmospheric monitoring. Third, we could not differentiate between respondents living in market-rate MUH and respondents living in subsidized MUH. Therefore, estimates of SHS incursions reported here may not accurately reflect the extent of incursions by MUH type. However, Gentzke et al reported no differences in the prevalence of past 12-month incursions in market-rate (50%) versus subsidized MUH (51%) in 6 US cities (16). Furthermore, our estimates controlled for income, and these overall estimates may account for possible differences in reported incursions between market-rate and subsidized MUH. Finally, although two-thirds of smokers living in MUH reported they would not prefer a complete ban on smoking in their building, the measure used to assess preferences for complete bans included exterior grounds as part of the prohibition. It is possible that a greater percentage of smokers would prefer complete bans for indoor areas only. That said, including exterior grounds as part of a complete ban ensures that SHS cannot infiltrate private residences from the outdoors and therefore must be considered as part of a comprehensive smoke-free MUH policy.

From 2013–2015, more than half of all smokers living in MUH allowed smoking in their homes in the United States, Canada, and the United Kingdom. Almost one-third of smokers living in MUH where smoking was not banned but who had personal smoking bans reported SHS incursions into their homes at least some of the time. Although recent smoke-free regulations implemented by the US Department of Housing and Urban Development banned smoking in public housing in 2018 (31), additional work is needed to eliminate SHS exposure in all MUH. The extent of involuntary SHS incursions was similar across the 3 countries studied. Even though comprehensive smoke-free policies have been implemented in all 3 countries, it is necessary to increase awareness of the dangers of SHS among MUH operators and residents to increase support for comprehensive MUH smoking bans in the United States and internationally.
